# Behavioral responses to encounter of fishing boats in wandering albatrosses

**DOI:** 10.1002/ece3.2677

**Published:** 2017-04-04

**Authors:** Julien Collet, Samantha C. Patrick, Henri Weimerskirch

**Affiliations:** ^1^ Centre d'Etudes Biologiques de ChizéUMR 7372 CNRS – Université La RochelleVilliers‐en‐BoisFrance; ^2^ University of LiverpoolLiverpoolUK

**Keywords:** competition, fisheries, foraging decisions, movement ecology, seabirds, vessel monitoring system

## Abstract

Animals are attracted to human food subsidies worldwide. The behavioral response of individuals to these resources is rarely described in detail, beyond chances of encounters. Seabirds for instance scavenge in large numbers at fishing boats, triggering crucial conservation issues, but how the response to boats varies across encounters is poorly known. Here we examine the behavioral response of wandering albatrosses (*Diomedea exulans*), equipped with GPS tags, to longline fishing boats operating near their colony for which we had access to vessel monitoring system data. We distinguish between encounters (flying within 30 km of a boat) and attendance behavior (sitting on the sea within 3 km of a boat), and examine factors affecting each. In particular, we test hypotheses that the response to encountered boats should vary with sex and age in this long‐lived dimorphic species. Among the 60% trips that encountered boats at least once, 80% of them contained attendance (but attendance followed only 60% of each single encounter). Birds were more attracted and remained attending longer when boats were hauling lines, despite the measures enforced by this fleet to limit food availability during operations. Sex and age of birds had low influence on the response to boats, except the year when fewer boats came fishing in the area, and younger birds were attending further from boats compared to older birds. Net mass gain of birds was similar across sex and not affected by time spent attending boats. Our results indicate albatrosses extensively attend this fishery, with no clear advantages, questioning impacts on foraging time budgets. Factors responsible for sex foraging segregation at larger scale seem not to operate at this fleet near the colony and are not consistent with predictions of optimal foraging theory on potential individual dominance asymmetries. This approach complements studies of large‐scale overlap of animals with human subsidies.

## 1 Introduction

Animals often are attracted by food sources generated by human activities (Oro, Genovart, Tavecchia, Fowler, & Martínez‐Abraín, 2013). Revealing the processes involved in these interactions can be key to improve the effectiveness of management measures. These food resources have contributed to the growth or maintenance of some populations, but also led to dependency on human activities (Bicknell, Oro, Camphuysen, & Votier, 2013; Bugoni, McGill, & Furness, 2010; Oro et al., 2013). In the case of seabirds scavenging on fishery discards, the poor nutritional value of this food can affect reproductive success (Grémillet et al., 2008, 2016; Tew Kai et al., 2013). Accidental captures (bycatch) and collisions also negatively affect population dynamics through increased mortality (Anderson et al., 2011; Weimerskirch, Brothers, & Jouventin, 1997). Bycatch is one of the primary causes of population declines for more than 30 seabird species (Croxall et al., 2012). Moreover, these effects on populations or even communities can be complex when there are individual differences in the susceptibility to interact with boats (e.g., Barbraud, Tuck, Thomson, Delord, & Weimerskirch, 2013; Mills & Ryan, 2005; Tuck et al., 2015; Votier et al., 2004, 2010). Understanding which species, populations, and/or individuals are more susceptible to interact with fishing boats, and why, is thus of primary concern for population predictions and management.

Within species, there can be important variations in the extent of bird–boat interactions between (Granadeiro, Phillips, Brickle, & Catry, 2011) and within populations (e.g., García‐Tarrasón et al., 2015; Granadeiro et al., 2011; Patrick et al., 2015; Votier et al., 2010). Individual variations in the extent of interaction can often be explained by individual variation in the overlap of foraging grounds with fishing areas. For instance, the frequent foraging sexual segregation of albatrosses and petrels worldwide can lead to sex‐biased bycatch in these species (Bugoni, Griffiths, & Furness, 2011; Weimerskirch et al., 1997). Nevertheless, evidence is accumulating that individuals close to vessels may not always end up scavenging at them (Bodey et al., 2014; Sugishita, Torres, & Seddon, 2015; Torres, Sagar, Thompson, & Phillips, 2013a). Understanding why some individuals stop or not at boats may thus be an important but mostly overlooked parameter to account for, in complement to large‐scale overlap assessment (Bodey et al., 2014; Croxall et al., 2013; Torres, Sagar, Thompson, & Phillips, 2013b).

In particular, dominance interactions may largely influence individual responses to boats and thus create variation in the risk of interaction between individuals overlapping over the same fleets. Indeed, inter‐ and intraspecies interference competition can be high when dense aggregations occur behind boats (Arcos, 2002; chap. 1; Cherel, Weimerskirch, & Duhamel, 1996; Furness, Ensor, & Hudson, 1992; Hudson & Furness, 1989). The assumption that competitive ability could affect attraction and response to boats is recurrent in the literature, but has rarely been tested (Arcos, Oro, & Sol, 2001; Bugoni et al., 2011; Ryan & Boix‐Hinzen, 1999; Weimerskirch, Salamolard, Sarrazin, & Jouventin, 1993). This is probably largely due to the challenges of on‐board observation conditions: The limited visual reach impedes the detection of nonattracted birds (Skov & Durinck, 2001); moreover, it can be difficult to distinguish and focus on single individuals for long periods of time, let alone identify its sex or age. Yet, we could expect from optimal foraging theory (OFT) that subdominant individuals would be less likely to join an aggregation at a boat when they find one (i.e., fly within attraction distance; Lee, Ounsley, Coulson, Rowcliffe, & Cowlishaw, 2016). They may also have lower energy yields (González‐Solís, Croxall, & Wood, 2000; Lee et al., 2016) or be relegated further from the actual source of food (i.e., stay further from boats when attending them; Parker & Sutherland, 1986).

Wandering albatrosses (*Diomedea exulans*) are known to widely attend fishing boats (e.g., Ashford, Croxall, Rubilar, & Moreno, 1995; Cherel et al., 1996) where they can dominate agonistic interactions over smaller‐sized species (Weimerskirch, Jouventin, & Stahl, 1986). They have suffered heavily from bycatch mortality worldwide (e.g., Nel et al., 2002; Otley et al., 2007; Weimerskirch et al., 1997), but little is known about individual variation in their interaction behavior with boats. Females are 20% smaller than males on average (Shaffer, Weimerskirch, & Costa, 2001), and when incubating, they tend to forage north of the colony, while males tend to go south where less fishing fleets operate (Weimerskirch et al., 2014). Competitive exclusion of smaller females by larger males has been suggested to explain these patterns (Weimerskirch et al., 1993; but see Shaffer et al., 2001; for an alternative hypothesis). In addition, reproductive performance varies with the age of individuals in this species (Froy, Phillips, Wood, Nussey, & Lewis, 2013; Pardo, Barbraud, & Weimerskirch, 2013; Weimerskirch, Lallemand, & Martin, 2005). It has been suggested that this could be related to changes in foraging areas and/or efficiency when aging (Lecomte et al., 2010; Patrick & Weimerskirch, 2015; Weimerskirch et al., 2014).

Here we examine the behavioral responses of wandering albatrosses from the Crozet Islands to the longline fishing fleet operating near the colony (seven boats in total). We used GPS‐tracking data collected over three consecutive breeding seasons on incubating birds of known age and sex, together with GPS positions of boats as recorded for the vessel monitoring system (VMS). This fine‐scale resolution data allowed us to define encounter events (birds remaining within attraction range of a boat, beyond on‐board observation scope) and attendance behavior (sitting within very close range of a boat), hence to evaluate encounter rates, probability to join an encountered boat compared to simply fly past, and several parameters of attendance behavior. We investigated first the extent of overlap and attendance of wandering albatrosses with the fishing fleet operating close to the colony, second whether these responses to boats differed between sex, age, and/or breeding season, and third what could be the consequences of attendance behaviors and their variation for the net mass gain of birds at sea.

## 2 Materials and Methods

### 2.1 Bird data

The study was carried out on Ile de la Possession (Crozet Archipelago 46°S, 52°E). In total, 160 incubating adult birds were equipped with GPS tags (IgotU Mobile Action Technology) in 2011, 2012, and 2013 between mid‐January and mid‐March.

Birds were caught on their nest and the GPS tags, encased in heat shrink tubing, were attached onto back feathers using adhesive Tesa tape. The total mass of attached devices (<32 g including the final package, 0.3%–0.5% of the bird body mass) was well under the 3% recommended threshold (Phillips, Xavier, Croxall, & Burger, 2003). Birds were recaptured on the nest after they left for at least one foraging trip. All GPS tags had a recording frequency of 15 min. In addition, 45 females (18 in 2011, 13 in 2012, and 14 in 2013) and 44 males (20 in 2011, 10 in 2012, and 14 in 2013) were weighed during both equipment of logger and recapture. As birds were not equipped during changeovers, we corrected these mass measurements to take into account rates of mass loss on the nest (Weimerskirch, 1995).

This population has been studied, and each individual banded since 1966 (Weimerskirch et al., 1997). For individuals that were not banded as chicks, we estimated their minimum age as the date of first capture plus 7 years, the youngest age of first breeding attempts (Weimerskirch et al., 1997). Sex was determined from a combination of size differences (Shaffer et al., 2001), copulation and plumage observations, and/or genetic analyses (Weimerskirch et al., 2005). For five individuals, we were uncertain of the age (*n* = 3) or sex (*n* = 2) and we removed them from the analyses.

For the remaining individuals, a total of 199 tracks were recorded, but only 194 occurred while at least one boat was present on the Crozet shelf (Table 1).

**Table 1:** Trip‐level statistics in relation to year, age and sex

	Total	Males			Females			Year		
		Total males	26+ years	<26 years	Total females	26+ years	<26 years	2011	2012	2013
Number of tracks (of individuals)	194 (156)	91 (71)	39 (30)	52 (41)	103 (85)	46 (38)	57 (47)	43 (43)	24 (24)	127 (107)
Max range (km)	1,052 ± 56	1,000 ± 97	1,002 ± 149	998 ± 128	1,098 ± 63	1,014 ± 95	1,165 ± 85	748 ± 102	977 ± 179	1,169 ± 69
Total distance covered (km)	4,685 ± 252	4,433 ± 399	4,418 ± 676	4,443 ± 487	4,907 ± 319	4,409 ± 423	5,310 ± 460	3,325 ± 406	4,537 ± 678	5,173 ± 328
Duration (days)	9.3 ± 0.3	8.7 ± 0.4	8.7 ± 0.7	8.7 ± 0.6	9.8 ± 0.5	10 ± 0.7	9.6 ± 0.7	9.1 ± 0.7	9.2 ± 0.8	9.4 ± 0.4
Average number of boats present during the trip	2.1 ± 0.1	2.2 ± 0.2	2.4 ± 0.3	2.0 ± 0.2	1.9 ± 0.1	2.0 ± 0.2	1.9 ± 0.2	4.0 ± 0.3	2.9 ± 0.0	1.2 ± 0.0
Proportion of trip over Crozet shelf (%)	40.2 ± 2.7	50.7 ± 4.2	53.6 ± 6.6	48.6 ± 5.5	30.9 ± 3.2	32.3 ± 5.2	29.8 ± 4.0	59.1 ± 5.8	42,0 ± 8.2	33.5 ± 3.1
% Of trips (# tracks. # individuals) with encounter event	60.3 (117, 100)	64.8 (59, 49)	69.2 (27, 23)	61.5 (32, 26)	56.3 (58, 51)	54.3 (25, 22)	57.9 (33, 29)	90.7 (39, 39)	87.5 (21, 21)	44.9 (57, 49)
% Of trips (# tracks. # individuals) attending boats when having the opportunity	77.8 (91, 81)	83.1 (49, 40)	92.6 (25, 22)	75.0 (24, 18)	72.4 (42, 41)	72.7 (18, 17)	77.1 (24, 24)	89.7 (35, 35)	66.7 (14, 14)	73.7 (42, 37)
Absolute time spent attending boats (hrs) during a trip, when >0	11.0 ± 1.0	9.7 ± 1.1	9.9 ± 1.7	9.5 ± 1.4	12.6 ± 1.6	15.3 ± 2.7	10.5 ± 2.0	12.5 ± 1.6	8.8 ± 2.4	10.5 ± 1.4
Proportion of trip spent attending boats (%) when >0	7.6 ± 0.7	7.5 ± 0.8	8.1 ± 1.4	6.9 ± 0.9	7.7 ± 1.1	9.0 ± 1.6	6.7 ± 1.5	8.1 ± 1.1	6.1 ± 1.8	7.7 ± 1.0

Note that age was treated as a continuous variable in all analyses. Statistics are means ± *SE* over all concerned trips.

### 2.2 Vessel data

We used data from VMS (boat GPS locations recorded every 1 hr) and fishing events (GPS points taken at the start and end of each line setting or hauling), both made available from the Pecheker database hosted at the Museum National d'Histoire Naturelle de Paris (Martin & Pruvost, unpublished data; Pruvost, Martin, Denys, & Causse, 2011). Following Collet, Patrick, and Weimerskirch (2015), we merged VMS data with fishing activity data to recreate trajectories, that were then linearly interpolated to estimate one point every 10 min. This 10‐min resolution means that all bird locations would fall within 5 min of a vessel location, while keeping a large proportion of noninterpolated vessel GPS positions (~1/3–1/5). All VMS points were categorized either as “transit” or “fishing” according to fishing operation records.

In 2011 and 2012, seven vessels were active over the study period (though not necessarily all simultaneously; range 0–6, Table 1). In 2013, only four vessels came, with some periods where there were no active boats over the Crozet area.

This fleet complies with mitigation measures aimed at reducing albatross bycatch. These include setting lines only at night, when albatrosses are much less active, such that most interactions occur when boats are hauling lines (see Section 2.1).

### 2.3 Behavioral modeling of the bird's response to boats

For each bird location, we determined simultaneous locations (±5 min) of each boat present and hence calculated the distance to each of the boats.

When this distance was less than 30 km, we considered the bird location within “attraction range,” that is, close enough to potentially detect and approach the boat. Indeed, data show that wandering albatrosses display flight movement directed toward boats more than expected by chance up to ca. 30 km, coinciding with the theoretical visual scope limit (Collet et al., 2015).

If the location was within 3 km, and with a speed <10 km/hr (indicative of a bird sitting on the water), the location was considered as “attending behavior,” with possible feeding attempts (albatrosses need to sit on the water to feed). Note that attendance behavior is necessarily within attraction range. This 3‐km value was chosen because wandering albatrosses were shown to sit on the water more than usual at distances up to 3 km from boats (Collet et al., 2015).

**Figure 1:**
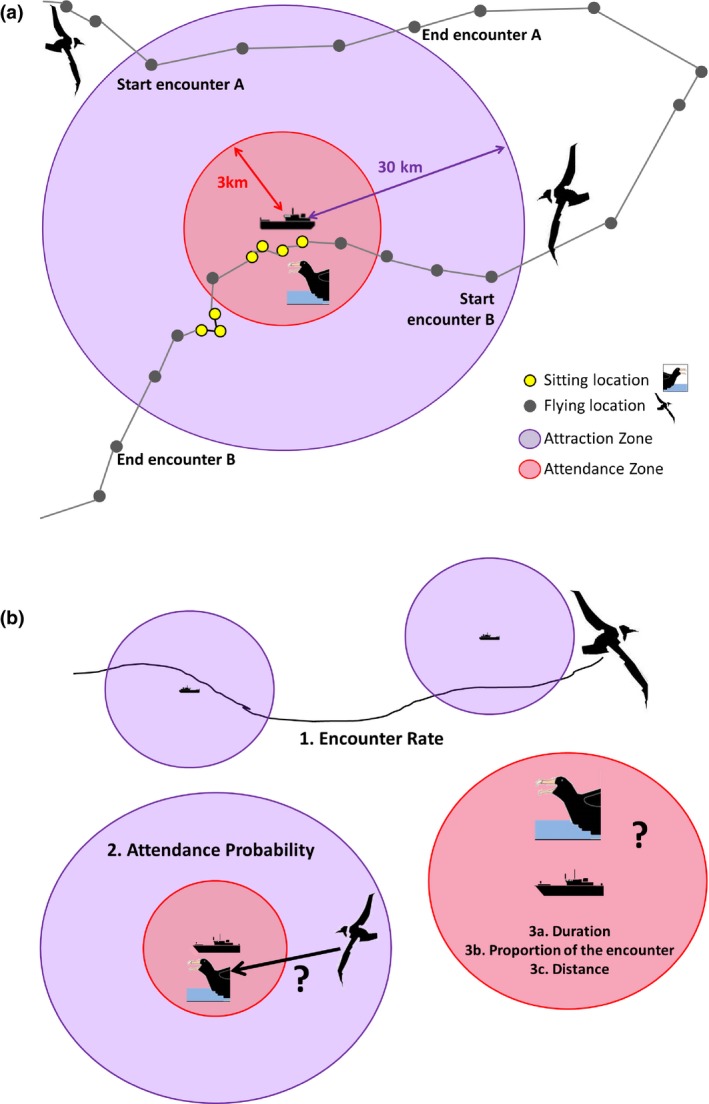
(a) Definitions used for modeling the behavioral response to boats (note that the circles actually move with the boat). Two hypothetical encounter events are depicted: (1) encounter without attendance, lasting 3 locations and (2) encounter followed by attendance, lasting 14 locations including four “attendance behavior” locations (speed <10 km/hr indicating sitting, in yellow, 29% of the encounter, at an average distance «3 km). There is a lag of six locs in between the two encounters, which is more than our threshold time‐to‐return value (four locs) so one and two are considered distinct encounters. (b) Models used in the analyses, following the definitions (see Table 2)

We defined an “encounter event” as a distinct series of consecutive locations that remain within attraction range (30 km) of at least one boat, without exiting this range for more than four consecutive GPS locations (ca. 1 hr, “time‐to‐return” parameter). Encounter events are defined independently of whether they contain attendance behavior (Figure 1a). This in turn enables us to model the behavioral response of birds to boats within the conceptual framework of OFT, considering the boat as a patch. The 1‐hr “time‐to‐return” value (i.e., allowing an exit of less than only four GPS locations) was chosen to limit assumptions on how long albatrosses can remember where previously encountered boats were, once having lost sight of them, while in the same time accounting for potential inaccuracies in bird–boat distances due to relatively low GPS acquisition frequency (at least one “true” boat position is recorded every hour).

Note that our choices for the attraction threshold and the time‐to‐return value limit “false negative” detections of encounter events, at the expense of potentially increasing false positive (i.e., no actual boat detection by the bird) or artificially splitting “true encounters.” We include a sensitivity analysis for these two parameters (15–30 km, 0.5–24 hr) in S.I.1 that shows our conclusions are very robust to this choice.

From this decomposition, we could first calculate encounter rates of birds with boats. Then, we assessed the proportion of encounter events that did contain attendance behavior. Assuming that other birds are already attending this boat when our tracked individual makes a decision whether to attend the boat (large number of seabirds indeed attend this fleet; Cherel et al., 1996), this probability to attend a boat is analogous to a scrounging probability within the producer–scrounger framework (Lee et al., 2016). Finally, for each encounter event containing attendance behavior, we could also determine how much time birds spent in attendance behavior (calculated either in absolute value or relative to the whole encounter event duration), analogous to a residence time, and how close to vessels they were on average when doing so (assuming more food is available closer to vessels; Collet et al., 2015).

Because wandering albatrosses are less active at night and their visual range is considerably limited at night or when they sit on the sea surface, we only considered in our analyses the 70.5% of encounter events that contained at least one position with speed >10 km/hr (i.e., “flying”) during daylight (day was defined by a solar elevation higher than 6° below the horizon). These retained encounter events included 93.5% of all locations classified as “attendance behavior.”

### 2.4 Statistical modeling of the factors influencing the behavioral response

We built five independent GLMMs (Figure 1b): one for encounter rate (number of encounters offset by trip duration, modeled with a negative binomial error structure), one for attendance probability at an encounter (binary response: attendance or not, modeled with a binomial error structure), and one for each of the parameters describing attendance behavior, average distance from boat (Gaussian error structure), duration (as the number of attending GPS locations in each encounter, modeled with a negative binomial error structure), and proportion of the encounter event spent attending (number of attending GPS locations offset by encounter duration, modeled with a negative binomial error structure). Response variables dealing with duration were accounted for by the relevant discrete number of GPS locations instead of absolute time value, because the distributions were largely zero‐skewed and more accurately modeled through Poisson‐like distributions than Gaussian distributions. We used negative binomial distributions to account for important overdispersion (Zuur, 2009).

Age and sex, and their interaction were included in all of the models. To account for large‐scale boat density effects, we included the concurrent average number of boats over the Crozet shelf during the bird trip in the encounter rate model. However, for all other models, we rather included year and the year–sex and year–age interactions, grouping 2011 and 2012 together against 2013, as there was consistently much less boats in the area in 2013 (in figures, we illustrate each year separately). Including year rather than actual concurrent boat density seemed more relevant to us for models of behavioral decisions, as we do not see how individuals could have accurate information on the concurrent boat density, but they could have gathered some on the current year conditions, from past trips' experience. In addition, for all models except that of encounter rates, we included as a covariate the average number of vessels present within 30 km of the bird during the encounter (indeed, in 11.0% of encounters, more than one boat at a time was within detection range). For all models except that of encounter rates, we also included a variable accounting for vessel fishing activity, and two different variables were calculated, one for attendance probability and one for attendance behavior parameters.

Sample sizes are given in Table 2. Random effects fitted were both trip ID and bird ID for all models except that of encounter rates, which is calculated at the trip level hence only included bird ID. First‐order interaction terms were removed from models when nonsignificant, but all fixed (and random) effects were maintained in final models.

**Table 2:** Model outputs for the behavioral responses to boats (model numbers refer to Figure 1b)

Model	Description		Influencing factors (estimators ± standard errors)							
	Population average value	*N* (encounters, tracks, individuals)	Average number of boats	Boat activity	Age	Sex (F vs. M)	Age:sex	Year 2013	Year:age	Year:sex
1. Encounter rate	0.30 ± 0.03 per day of trip	*,194, 156	+0.45 ± 0.04		NS	NS	NS			
2. Probability to interact at an encounter	60.5%	390, 117, 100		+0.19 ± 0.07	NS	NS	NS	NS	NS	NS
3a. Time spent attending boat per encounter	4.34 ± 0.29 hr	236, 91, 81		+2.17 ± 0.33	NS	+0.39 ± 0.13	NS	NS	NS	NS
3b. Proportion of the encounter spent attending boats	28.8% ± 1.0%	236, 91, 81		+0.67 ± 0.24	NS	+0.21 ± 0.10	NS	−0.19 ± 0.09	NS	NS
3c. Average distance from boats when attending them, per encounter	1,254 ± 34 m	236, 91, 81		−569 ± 192	+5.88 ± 5.43	NS	NS	+656 ± 204	−18.88 ± 7.92	NS

Effects of the number of boats present within 30 km are indicated in the Section 2.1 and not reported here. Estimated ± *SE* are given when they were significant. N.S. for nonsignificant.

#### 2.4.1 Accounting for vessel fishing activity

In the case of the attendance probability at an encounter, it is the minimum time elapsed between the start of the encounter event (when the bird enters the 30‐km circle) and either the end of the last fishing operations or the start of the next fishing operations (a fishing operation is either line setting or line hauling). This variable will be 0 if the vessel is fishing when the bird enters within 30 km of the boat, but could be for instance 30 min if the vessel stopped fishing half an hour before the encounter, or started fishing half an hour after the start of the encounter (Figure 3). This measure allows us to directly compare encounters with or without attendance behavior.

For all three models of attendance behavior, we accounted for the vessel activity by including the proportion of the encounter event with the vessel being in active fishing operations.

### 2.5 Mass gain analyses

We modeled the mass gained at sea (g) in function of year, sex, and level of attendance to boats (the latter proportional to total trip duration). To test the hypothesis that females would obtain less offal at boats because of competition with larger males, we also included the interaction between sex and level of attendance to boats. Following results from Cornioley, Börger, Ozgul, and Weimerskirch (2016), we also included mass at departure as a covariate (using within‐sex anomalies in mass at departure to account for size dimorphism). As we had no repeat measures for individuals, we used a linear model without random effects. Similar results were obtained when looking at mass gain rates (g/day at sea).

All analyses were conducted in the R environment. Codes are publically available at https://github.com/julco134/birboat, along with a subsample of relevant data.

## 3 Results

**Figure 2:**
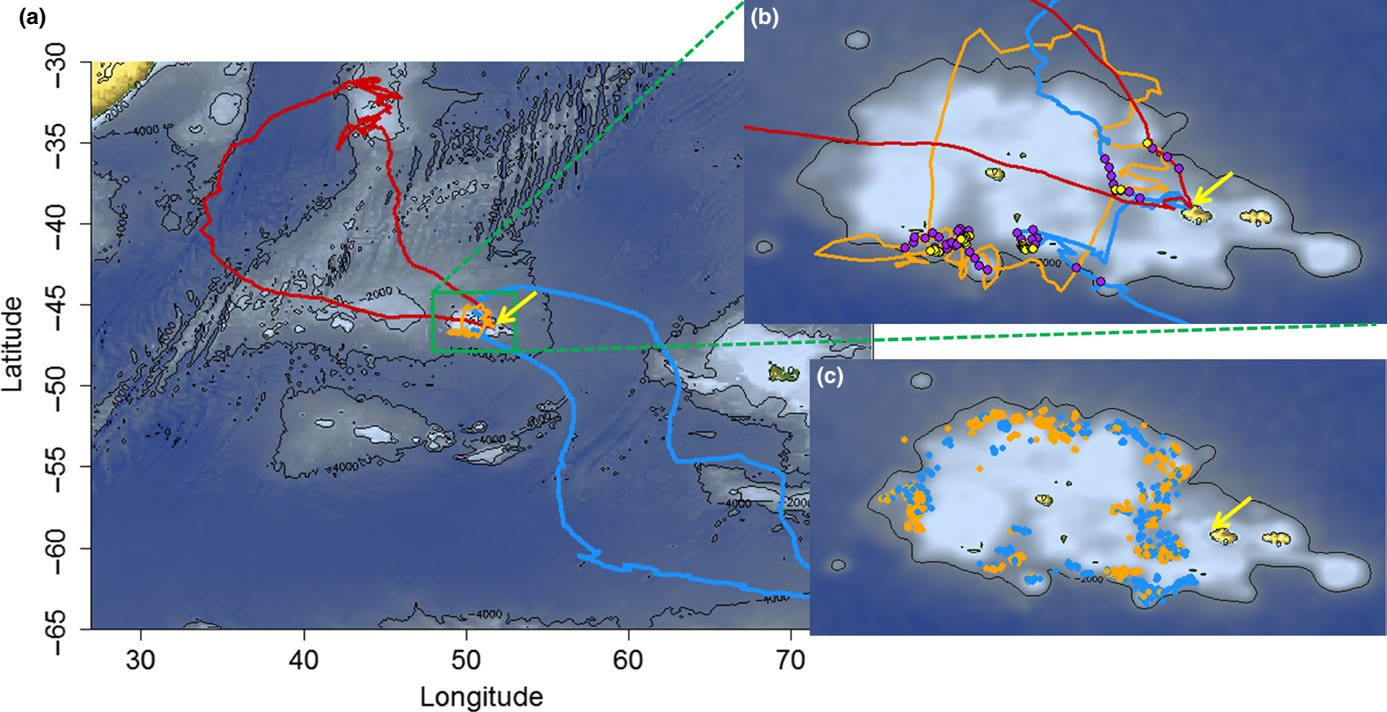
(a) Three typical foraging trips of wandering albatrosses from Crozet (yellow arrow). Two mostly oceanic trips, one from a female (in red, 15.5 days) and one from a male (in blue, 19.3 days), and one trip (in orange, from a female, 3.9 days) remaining over the Crozet shelf. (b) Zooms over the Crozet shelf. Locations in flight within 30 km of a boat shown in purple, and locations sitting within 3 km of a boat (attendance) shown in yellow. (c) All locations classified as attendance behavior for males (blue) and females (orange)

Foraging trips varied extensively in duration or distance travelled (194 trips ranging in duration from 2 to 29 days; see Table 1 for more details). All bird trips passed over the Crozet shelf to either reach oceanic waters or stay on the shelf edge. Some trips were mainly restricted within this shelf (Figure 2a,b), but most contained oceanic portions, to variable extents (average 40% of time over the shelf; see Table 1, Figure 2a,b). Females tended to have longer trips in our sample, but the difference was not significant (*t* = −1.560, *p* = .12), and they spent proportionally less time over the shelf (−20.0% ± 5.3%, *t* = 3.790, *df* = 172, *p* < .001; Table 1), but did not spend less time attending boats (*z* = −0.10, *p* = .99; Table 1). Boat activity was restricted to the Crozet shelf edge, with boats transiting between different areas within it (Figure 2c).

### 3.1 Encounter rate and probability to attend boats at encounters

About 60.3% of the 194 bird foraging trips passed at least once within 30 km of a boat during daytime (i.e., ≥1 “encounter event”). Attendance behavior (sitting within 3 km of a boat) occurred in only 60.5% of encounter events (236 of 390), but because trips could contain several encounters, attendance behavior eventually occurred in 79.5% of these trips with at least one encounter (Table 1). Attendance behavior represented 7.6% ± 0.7% of total trip duration on average (max 24.5%; Table 1) for birds attending at least once a boat.

Only the average number of boats present in the Crozet sector during the trip had a significant, positive effect on encounter rate (*z* = 6.231, *p* < .001; Table 2). Females and males had similar encounter rates (Table 2), and they attended boats over the same areas within the shelf (Figure 2c).

**Figure 3:**
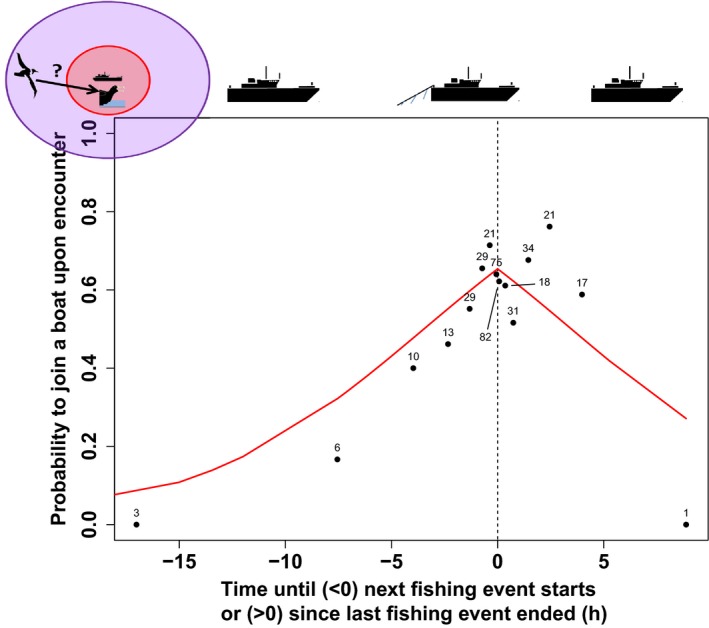
Observed (filled dots) and modeled (red solid line) probability to attend a boat upon encounter, depending on time to closest fishing activity at the start of the encounter. Numbers of observations over which proportions were calculated are indicated above each dot. Dashed line shows when the boat was active at the start of the encounter

Age, sex, and year had no influence on the probability to attend after encounter (Table 2), but birds encountering transiting boats were less likely to attend boats than those encountering boats in fishing operation (*z* = −2.580, *p* < .001; Table 2, Figure 3). Birds were more likely to be attracted when several boats were within detection range (3.62 ± 1.29, *z* = 2.807, *p* < .01).

### 3.2 Behavior while attending boats

The higher the fishing activity of the vessel during an encounter event, the longer in absolute time (*z* = 12.43, *p* < .001) or proportional time (*z* = 2.828, *p* < .01) birds spent attending it (Table 2). Birds attended at closer distances from the boat when boats were active (χ^2^ = 8.800, *df* = 1, *p* < .01; Table 2). The more the boats within 30 km, the more time remaining attending a boat at an encounter (0.80 ± 0.25, *z* = 3.188, *p* = .001), but it had no effect on the average distance from boats when attending (χ^2^ = 0.157, *df* = 1, *p* = .69), and there was a tendency to spend a lower proportion of the event attending (−0.32 ± 0.17, *z* = −1.858, *p* = .06) when more boats were present.

Hence at the population level, attendance behavior occurred mainly when boats were hauling lines (66.3% of all attending locations, day or night) or when no fishing operations were ongoing (31.5%) but rarely during line setting (2.1%). As a comparison, 56.3% of nonattending locations that were within 30 km of a boat occurred while no fishing operations were ongoing, 34.0% occurred during line hauling, and 9.6% during line setting.

**Figure 4:**
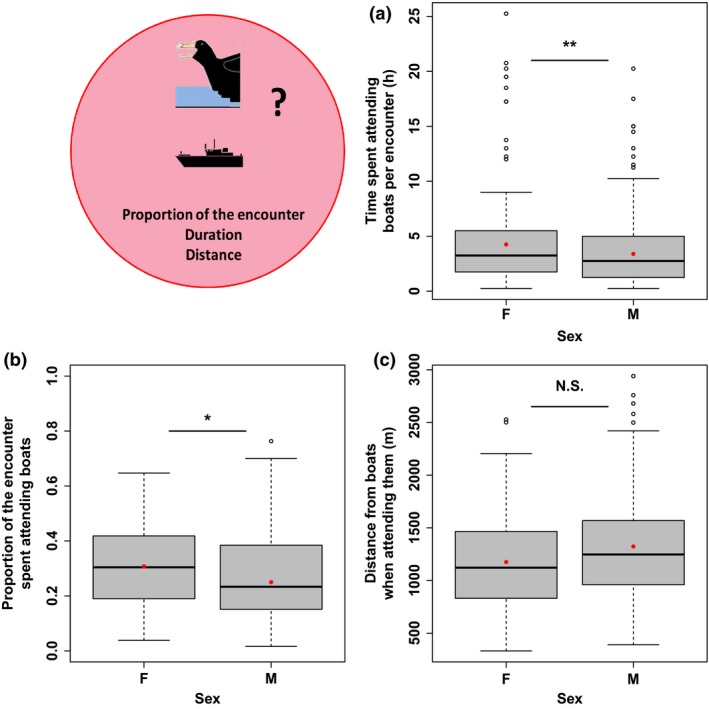
Sex effects on attendance behavior: duration (a), proportion of the whole encounter event (b), and average distance from boats (c). Modeled means (Table 2) are shown as red dots

Compared to males, females spent more time attending boats at each encounter event both in absolute value (*z* = −2.921, *p* < .01; Table 2) and in proportion to the encounter duration (*z* = −2.140, *p* = .03), although the differences appear rather small (Figure 4a,b). Females were not further from boats when attending them (Figure 4c, Table 2).

**Figure 5:**
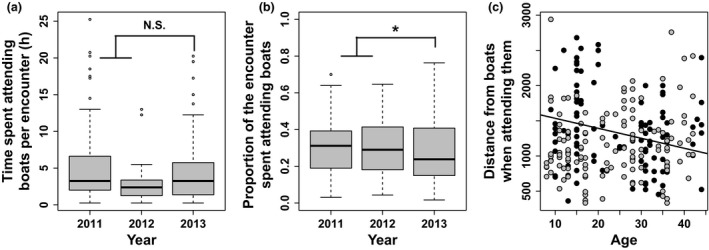
Year effects on attendance behavior: duration (a), proportion of the whole encounter events (b), and distance from boats (c). The year effect depended on the age of birds in 2013 (black dots, regression line drawn), but not in 2011–2012 (gray dots)

Fewer vessels were active in 2013 compared to 2011 and 2012 (Table 1). In 2013, birds stayed the same absolute amount of time attending boats during each encounter as in other years (Table 2, Figure 5a). However, they spent proportionally more time out of the “attending area” during an encounter (*z* = −2.372, *p* = .02; Figure 5b), and on average stayed further from vessels when attending them (+656 ± 204 m; Figure 5c).

Age had no effect on the attendance behavior (Table 2), except in 2013 when younger birds were further from boats when attending them, compared to older birds (χ^2^ = 5.815, *df* = 1, *p* = .02, Figure 5c).

### 3.3 Mass gained at sea

Birds with comparatively lower mass at departures (accounting for sex dimorphism) had higher absolute mass gains (*t* = −5.821, *df* = 85, *p* < .001).

**Figure 6:**
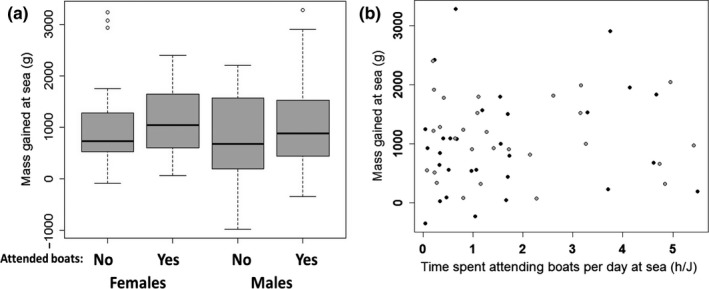
Net mass gained at sea by males (in black in B) and females (gray in B), whether or not they attend boats (a), and for those that did attend boats, in function of the proportion of their time at sea spent attending boats (b)

Whether birds attended boats or not during their trip had no effects on their mass gain (*t* = 0.886, *df* = 85, *p* = .38; Figure 6a) without interaction with sex (*t* = 0.858, *df* = 84, *p* = .39, Figure 6a). Actually, males and females did not differ in mass gained at sea (*t* = −1.091, *df* = 85, *p* = .28). Mass gains were similar across years (*t* < 0.601, *df* = 85, *p* > .55).

When examining birds attending boats, the proportion of the total trip duration spent attending boats did not influence the mass gained at sea (*t* = 0.162, *df *= 52, *p* = .87; Figure 6b). Again, there were no sex effects (*t* = −0.475, *df *= 52, *p* = .64, Figure 6b), nor interaction between sex and time spent attending boats (*t* = 0.370, *df* = 51, *p* = .71), and no differences between years (*t* < 0.901, *df* = 52, *p* > .37).

## 4 Discussion

Our study is the first to decompose the behavioral response of seabirds to boats into encounter probability, attraction probability after encounter, and attendance behavior once at boats, and to relate each of these steps to boat activity or individual characteristics. Our results show that 60% of individuals of any age or sex encountered one or more boat, and 80% of them attended at least one of the boat encountered. Hence overall, nearly 50% of the birds tracked attended fishing vessels in the Crozet sector, and this number would have been much higher were it not a peculiar year of low boat presence (2013) that limited encounter rates. These results provide support for using individual large‐scale foraging range overlap with boats as a reasonable proxy for interaction risks in this species (Croxall et al., 2013; Jiménez et al., 2015). Nevertheless, we add on growing evidence that a significant proportion of encounters (~60% here) are not immediately followed by interactions (Bodey et al., 2014; Sugishita et al., 2015; Torres et al., 2013a), suggesting that caution is required to derive precise quantitative prediction of interaction risk from mere overlap data (Croxall et al., 2013; Torres et al., 2013a,b).

Potential dominance effects mediated by sexual size dimorphism or age had a limited influence on behavioral response to boats. In particular, the sex or age foraging segregations observed at larger spatial scales in this species (Weimerskirch et al., 2014) did not operate at the scale of fleet attendance, with the exception of younger birds relegated further from boats in 2013 when less vessels were present overall. The modeling approach developed here allows the examination of behavioral decisions of animals exploiting human‐generated resources using the predictions of optimal foraging theory. OFT predicts that foraging decisions (when to join a patch, how long to exploit a patch, and how to distribute among and/or within patch) will differ with individual dominance (e.g., Lee et al., 2016; Parker & Sutherland, 1986). The strong size dimorphism between sexes in this species (Shaffer et al., 2001) is often assumed to lead to such sexual dominance asymmetry (González‐Solís et al., 2000; Ryan & Boix‐Hinzen, 1999; Weimerskirch et al., 1993) and thus to different behavioral responses to boats and/or sexual segregation at boats, as is observed at larger scale (Table 1; Weimerskirch et al., 1993, 2014). Our results show the opposite: Females and males attended boats in the same areas, had similar encounter rates with boats, and had the same probability to join a boat at an encounter. Females spent slightly higher amounts of time attending boats at each encounter than males and were not relegated further from boats when attending them. Hence, we found no evidence for competitive exclusion by larger males at boats. As a matter of fact, overall, females spent more time attending this fleet during their trip in terms of absolute time. Finally, males and females had similar mass gain, which was not influenced by the time spent attending boats. To our knowledge, the size‐mediated competitive exclusion hypothesis has been reliably established in only one case for seabirds (González‐Solís et al., 2000). The fact that it is not observed in wandering albatross, a highly dimorphic species, questions whether such intraspecific size dominance plays a significant role in seabird–fisheries interactions, and hence would affect how individuals distribute at boats when they overlap with the same fleets. Further work on other species is nevertheless required as wandering albatrosses both occupy a distinct position in seabird aggregations, where they dominate all other, smaller‐sized species (Weimerskirch et al., 1986), and seem to be much less active at feeding aggregations compared to other albatrosses (Ashford et al., 1995; Cherel et al., 1996). Hence, the effects of intraspecific dominance may be lower in wandering albatrosses than for other species.

The availability of boats around the island influenced the relationships we found. When boat density was higher, encounter rates were higher. Moreover, when there were fewer boats available around the islands in 2013, attending birds were overall further from vessels than in other years and spent a lower proportion of time attending boats at each encounter (i.e., spent more time off the 3‐km area while still remaining close to boats). Nevertheless, the absolute value of time spent attending boats at each encounter was similar across years, as was the attraction probability. This might be the result of an increased spatial competition if a similar number of seabirds concentrated around a fewer number of vessels. The 2013 effect on the attendance distance was most marked in younger rather than older birds (Figure 5c), suggesting that experience might play a role on the efficiency of individuals to position within aggregations when bird density increases because of lower boat density. This may be of importance as collisions or bycatch is more likely to occur closer to the boat rather than away when lines are in deeper waters. However, we stress that further work is needed to confirm this age pattern.

As sex and age were poor predictors, a large part of the variation in the attendance behavior remains to be explained. The value of 30 km we used for the attraction range may be an upper limit to the visual detection and therefore not always realized (Collet et al., 2015), so that birds may simply not have detected boats in most possible encounters (Weimerskirch, Capdeville, & Duhamel, 2000). Using more complex modeling approach to try estimating separately detection probability from attraction probability after detection, and including data on wind and/or weather, may be the way forward. The probability to be attracted increased when adopting more restrictive encounter definitions (such as shorter attraction range thresholds), but even then a non‐negligible proportion of encounters were not “exploited” by birds (S.I.1).

We nevertheless found that a boat engaged in fishing operations was more likely to attract birds, and that birds stayed attending the boat for longer when these were actively fishing. This is despite the mitigation measures that have been implemented since the mid‐2000 in the Crozet exclusive economic zone to reduce bycatch. These measures include the setting of lines only at night when diurnal albatrosses rest at the sea surface (explaining why attendance occurred mainly during hauling and why we excluded night locations for our analyses), the use of weighted lines for faster sinking speeds (wandering albatrosses are strict surface feeders), and ban of any discarding during fishing operations: Fishermen have to delay it to the end of operations, when possible even after a block of several neighboring lines have been hauled, and to favor nonfishing areas for doing so (J.O. des T.A.A.F. 2010, sec. Annexe II‐Exercice de la pêche, and more recent ones).

The presence of government observers on‐board each boat ensures that these measures are effectively enforced. Although these measures are extremely efficient to eliminate albatross bycatch by this fleet (approximately null in the past decade; Delord, Gasco, Weimerskirch, Barbraud, & Micol, 2005), our results suggest they may not diminish bird attraction to boats. Even more, it questions why birds keep attending boats, especially during fishing operations, while supposedly much less food is available to them. Either they benefit from the presence of subsurface feeders such as diving petrels (Jiménez, Domingo, Abreu, & Brazeiro, 2012) and/or depredating killer and sperm whales (Tixier et al., 2010), that could facilitate access to baits or captures to surface feeders, either a large proportion of the time spent attending boats actually is not food rewarded (Ashford et al., 1995; Cherel et al., 1996). While it seemed not to have impacts on mass gain, such a behavioral trap could affect time budgets and cause issues for chick‐rearing birds with higher constraints on time and energy (see also Grémillet et al., 2008, 2016 for impacts of low food quality at boats). The facts that most individuals in our study may have been born before the implementation of these measures, and/or that this species can encounter other fleets further in their range, including during their sabbatical years, may help explain the persistence of this attraction during operations. Yet, there is anecdotal evidence that wandering albatrosses can quickly alter their foraging behavior after a change in food supplies (Gain, 1914 cited by Tickell, 2000), and our results indeed support some behavioral flexibility.

Another unknown aspect is how the presence of boats on the Crozet shelf affects wandering albatross foraging behavior at larger scales (Tew Kai et al., 2013). Fishing activity in the Crozet area started in the late 1990s (Pruvost, Duhamel, Gasco, & Palomares, 2015), and incubating birds from this colony exploited the shelf slope area before the commencement of fisheries; however, the extent of this behavior at this time is unclear (Weimerskirch et al., 1993). We show here that the proportion of time attending this fleet had no effects on the mass gained at sea, and represented on average less than 8% of the time spent at sea, so that individuals from this colony can still largely rely on other food resources. Determining whether the encounter rates are too high to be opportunistic rather than the result of an active searching process is difficult.

To conclude, we show here that wandering albatrosses attended extensively the fishing fleet operating close to the breeding grounds. Individuals of all age and sex had similar encounter rates with this fleet and attended it in similar proportions, except when resources were scarce and younger birds appeared to be attending further from boats than older birds. Given the strong age and sex patterning found in the foraging behavior of this species at larger scale, our results suggest that segregation is limited at boats accessible to all individuals, supporting the use of overlap data to assess risks of encounters in this species. Further work is needed to determine whether this applies to other species or fisheries, where competition intensity may be different. We identified vessel operations as a factor affecting both attraction probability and time spent attending behind boats, despite enforced measures to reduce boat attractiveness. This may have unforeseen consequences on time budgets if boats act as poorly rewarding foraging cues, and it highlights that mitigation measures designed for limiting bycatch will probably have a limited impact for the reduction of seabird behavioral dependency on boats when it occurs. Furthermore, we add on growing evidence that a large proportion of encounters are not immediately followed by attending behavior, calling for caution when trying to derive quantified interaction probabilities from large‐scale overlap data. Our results suggest that detection limits rather than bird decision‐making may be involved in our case, although this will require further investigation. However, we stress that when encounter rates are no longer limiting factors (high boat density and/or predictability), the relationship between overlap and interaction levels may be far from linear. Finally, while we developed this approach in the context of seabirds–fisheries interactions, it could be adapted for other human activities attracting wildlife, or to gain fundamental insights into wild animal decision‐making and/or detection capabilities.

## Conflict of Interest

None declared.

## Data Archiving

A subset of the data is available with all relevant R codes at https://github.com/julco134/birboat. More data could be shared on request, but there is restriction on sharing vessel‐related data.

## References

[ece32677-bib-0001] Anderson, O. R. , Small, C. J. , Croxall, J. P. , Dunn, E. K. , Sullivan, B. J. , Yates, O. , & Black, A. (2011). Global seabird bycatch in longline fisheries. Endangered Species Research, 14, 91–106.

[ece32677-bib-0002] Arcos, J. M. (2002). Foraging ecology of seabirds at sea: Significance of commercial fisheries in the NW Mediterranean. Universitat de Barcelona.

[ece32677-bib-0003] Arcos, J. , Oro, D. , & Sol, D. (2001). Competition between the yellow‐legged gull *Larus cachinnans* and Audouin's gull *Larus audouinii* associated with commercial fishing vessels: The influence of season and fishing fleet. Marine Biology, 139, 807–816.

[ece32677-bib-0004] Ashford, J. R. , Croxall, J. P. , Rubilar, P. S. , & Moreno, C. A. (1995). Seabird interactions with longlining operations for *Dissostichus eleginoides* around South Georgia, April to May 1994. CCAMLR Science, 2, 111–121.

[ece32677-bib-0005] Barbraud, C. , Tuck, G. N. , Thomson, R. , Delord, K. , & Weimerskirch, H. (2013). Fisheries bycatch as an inadvertent human‐induced evolutionary mechanism. PLoS One, 8(4), e60353. doi: 10.1371/journal.pone.0060353 23593199 10.1371/journal.pone.0060353 PMC3622665

[ece32677-bib-0006] Bicknell, A. W. J. , Oro, D. , Camphuysen, K. C. J. , & Votier, S. C. (2013). Potential consequences of discard reform for seabird communities. Journal of Applied Ecology, 50, 649–658. doi: 10.1111/1365‐2664.12072

[ece32677-bib-0007] Bodey, T. W. , Jessopp, M. J. , Votier, S. C. , Gerritsen, H. D. , Cleasby, I. R. , Hamer, K. C. , … Bearhop, S. (2014). Seabird movement reveals the ecological footprint of fishing vessels. Current Biology, 24, R514–R515. doi: 10.1016/j.cub.2014.04.041 24892908 10.1016/j.cub.2014.04.041

[ece32677-bib-0008] Bugoni, L. , Griffiths, K. , & Furness, R. (2011). Sex‐biased incidental mortality of albatrosses and petrels in longline fisheries: Differential distributions at sea or differential access to baits mediated by sexual size dimorphism? Journal of Ornithology, 152, 261–268. doi: 10.1007/s10336‐010‐0577‐x

[ece32677-bib-0009] Bugoni, L. , McGill, R. A. , & Furness, R. W. (2010). The importance of pelagic longline fishery discards for a seabird community determined through stable isotope analysis. Journal of Experimental Marine Biology and Ecology, 391, 190–200.

[ece32677-bib-0010] Cherel, Y. , Weimerskirch, H. , & Duhamel, G. (1996). Interactions between longline vessels and seabirds in Kerguelen waters and a method to reduce seabird mortality. Biological Conservation, 75, 63–70.

[ece32677-bib-0011] Collet, J. , Patrick, S. C. , & Weimerskirch, H. (2015). Albatrosses redirect flight towards vessels at the limit of their visual range. Marine Ecology Progress Series, 526, 199–205. doi: 10.3354/meps11233

[ece32677-bib-0012] Cornioley, T. , Börger, L. , Ozgul, A. , & Weimerskirch, H. (2016). Impact of changing wind conditions on foraging and incubation success in male and female wandering albatrosses. Journal of Animal Ecology, 85, 1318–1327. doi: 10.1111/1365‐2656.12552 27187714 10.1111/1365-2656.12552

[ece32677-bib-0013] Croxall, J. P. , Butchart, S. H. M. , Lascelles, B. , Stattersfield, A. J. , Sullivan, B. , Symes, A. , & Taylor, P. (2012). Seabird conservation status, threats and priority actions: A global assessment. Bird Conservation International, 22, 1–34. doi: doi: 10.1017/S0959270912000020

[ece32677-bib-0014] Croxall, J. , Small, C. , Sullivan, B. , Wanless, R. , Frere, E. , Lascelles, B. , … Yates, O. (2013). Appropriate scales and data to manage seabird‐fishery interactions: Comment on Torres et al. (2013). Marine Ecology Progress Series, 493, 297–300. doi: 10.3354/meps10599

[ece32677-bib-0015] Delord, K. , Gasco, N. , Weimerskirch, H. , Barbraud, C. , & Micol, T. (2005). Seabird mortality in the Patagonian toothfish longline fishery around Crozet and Kerguelen Islands, 2001–2003. CCAMLR Science, 12, 53–80.

[ece32677-bib-0016] Froy, H. , Phillips, R. A. , Wood, A. G. , Nussey, D. H. , & Lewis, S. (2013). Age‐related variation in reproductive traits in the wandering albatross: Evidence for terminal improvement following senescence. Ecology Letters, 16, 642–649. 23438213 10.1111/ele.12092

[ece32677-bib-0017] Furness, R. W. , Ensor, K. , & Hudson, A. V. (1992). The use of fishery waste by gull populations around the British Isles. Ardea, 80, 105–113.

[ece32677-bib-0018] Gain, L. (1914). Oiseaux antarctiques In Cie Masson (Eds.,), Deuxieme Expedition Antarctique Française—Sciences Naturelles (pp. 1–200). Paris, France: Masson et cie.

[ece32677-bib-0019] García‐Tarrasón, M. , Bécares, J. , Bateman, S. , Arcos, J. M. , Jover, L. , & Sanpera, C. (2015). Sex‐specific foraging behavior in response to fishing activities in a threatened seabird. Ecology and Evolution, 5, 2348–2358. doi: 10.1002/ece3.1492 26120425 10.1002/ece3.1492 PMC4475368

[ece32677-bib-0020] González‐Solís, J. , Croxall, J. P. , & Wood, A. G. (2000). Sexual dimorphism and sexual segregation in foraging strategies of northern giant petrels, *Macronectes halli*, during incubation. Oikos, 90, 390–398.

[ece32677-bib-0021] Granadeiro, J. P. , Phillips, R. A. , Brickle, P. , & Catry, P. (2011). Albatrosses following fishing vessels: How badly hooked are they on an easy meal? PLoS One, 6, e17467. doi: 10.1371/journal.pone.0017467 21399696 10.1371/journal.pone.0017467 PMC3047564

[ece32677-bib-0022] Grémillet, D. , Péron, C. , Kato, A. , Amélineau, F. , Ropert‐Coudert, Y. , Ryan, P. G. , & Pichegru, L. (2016). Starving seabirds: Unprofitable foraging and its fitness consequences in Cape gannets competing with fisheries in the Benguela upwelling ecosystem. Marine Biology, 163, 1–11. doi: 10.1007/s00227‐015‐2798‐2

[ece32677-bib-0023] Grémillet, D. , Pichegru, L. , Kuntz, G. , Woakes, A. G. , Wilkinson, S. , Crawford, R. J. , & Ryan, P. G. (2008). A junk‐food hypothesis for gannets feeding on fishery waste. Proceedings of the Royal Society B‐Biological Sciences, 275, 1149–1156. doi: 10.1098/rspb.2007.1763 10.1098/rspb.2007.1763 PMC2602693 18270155

[ece32677-bib-0024] Hudson, A. V. , & Furness, R. W. (1989). The behaviour of seabirds foraging at fishing boats around Shetland. Ibis, 131, 225–237.

[ece32677-bib-0025] Jiménez, S. , Domingo, A. , Abreu, M. , & Brazeiro, A. (2012). Bycatch susceptibility in pelagic longline fisheries: Are albatrosses affected by the diving behaviour of medium‐sized petrels? Aquatic Conservation: Marine and Freshwater Ecosystems, 22, 436–445.

[ece32677-bib-0026] Jiménez, S. , Domingo, A. , Brazeiro, A. , Defeo, O. , Wood, A. G. , Froy, H. , … Phillips, R. A. (2015). Sex‐related variation in the vulnerability of wandering albatrosses to pelagic longline fleets. Animal Conservation, 19, 281–295.

[ece32677-bib-0027] Journal Officiel des Terres Australes et Antarctiques Françaises (2010). Arrêté n° 2010‐53 du 30 août 2010 prescrivant les règles encadrant l'exercice de la pêche à la légine (Dissostichus eleginoides), aux raies (Bathyraja eatonii, Bathyraja irrasa, Raja taaf), au grenadier (Macrourus carinatus), autorisée dans les zones économiques exclusives de Crozet et de Kerguelen.

[ece32677-bib-0028] Lecomte, V. J. , Sorci, G. , Cornet, S. , Jaeger, A. , Faivre, B. , Arnoux, E. , … Weimerskirch, H. (2010). Patterns of aging in the long‐lived wandering albatross. Proceedings of the National Academy of Sciences, 107, 6370–6375. doi: 10.1073/pnas.0911181107 10.1073/pnas.0911181107 PMC2852007 20308547

[ece32677-bib-0029] Lee, A. E. G. , Ounsley, J. P. , Coulson, T. , Rowcliffe, J. M. , & Cowlishaw, G. (2016). Information use and resource competition: An integrative framework. Proceedings of the Royal Society of London. Series B: Biological Sciences, 283, 20152550. doi: 10.1098/rspb.2015.2550 26888031 10.1098/rspb.2015.2550 PMC4810826

[ece32677-bib-0031] Mills, M. S. , & Ryan, P. G. (2005). Modelling impacts of long‐line fishing: What are the effects of pair‐bond disruption and sex‐biased mortality on albatross fecundity? Animal Conservation, 8, 359–367.

[ece32677-bib-0032] Nel, D. C. , Ryan, P. G. , Nel, J. L. , Klages, N. T. W. , Wilson, R. P. , Robertson, G. , & Tuck, G. N. (2002). Foraging interactions between Wandering Albatrosses *Diomedea exulans* breeding on Marion Island and long‐line fisheries in the southern Indian Ocean. Ibis, 144, E141–E154. doi: 10.1046/j.1474‐919X.2002.00092.x

[ece32677-bib-0033] Oro, D. , Genovart, M. , Tavecchia, G. , Fowler, M. S. , & Martínez‐Abraín, A. (2013). Ecological and evolutionary implications of food subsidies from humans. Ecology Letters, 16, 1501–1514. doi: 10.1111/ele.12187 24134225 10.1111/ele.12187

[ece32677-bib-0034] Otley, H. , Reid, T. , Phillips, R. , Wood, A. , Phalan, B. , & Forster, I. (2007). Origin, age, sex and breeding status of wandering albatrosses (*Diomedea exulans*), northern (*Macronectes halli*) and southern giant petrels (*Macronectes giganteus*) attending demersal longliners in Falkland Islands and Scotia Ridge waters, 2001–2005. Polar Biology, 30, 359–368. doi: 10.1007/s00300‐006‐0192‐8

[ece32677-bib-0035] Pardo, D. , Barbraud, C. , & Weimerskirch, H. (2013). Females better face senescence in the wandering albatross. Oecologia, 173, 1283–1294. doi: 10.1007/s00442‐013‐2704‐x 23797411 10.1007/s00442-013-2704-x

[ece32677-bib-0036] Parker, G. A. , & Sutherland, W. J. (1986). Ideal free distributions when individuals differ in competitive ability: Phenotype‐limited ideal free models. Animal Behaviour, 34, 1222–1242. doi: 10.1016/S0003‐3472(86)80182‐8

[ece32677-bib-0037] Patrick, S. C. , Bearhop, S. , Bodey, T. W. , Grecian, W. J. , Hamer, K. C. , Lee, J. , & Votier, S. C. (2015). Individual seabirds show consistent foraging strategies in response to predictable fisheries discards. Journal of Avian Biology, 46, 431–440. doi: 10.1111/jav.00660

[ece32677-bib-0038] Patrick, S. C. , & Weimerskirch, H. (2015). Senescence rates and late adulthood reproductive success are strongly influenced by personality in a long‐lived seabird. Proceedings of the Royal Society of London. Series B: Biological Sciences, 282, 20141649. doi: 10.1098/rspb.2014.1649 25473008 10.1098/rspb.2014.1649 PMC4286031

[ece32677-bib-0039] Phillips, R. A. , Xavier, J. C. , Croxall, J. P. , & Burger, A. E. (2003). Effects of satellite transmitters on albatrosses and petrels. The Auk, 120, 1082–1090. doi: 10.1642/0004‐8038(2003) 120[1082:EOSTOA]2.0.CO;2

[ece32677-bib-0040] Pruvost, P. , Duhamel, G. , Gasco, N. , & Palomares, M. L. D. (2015). A short history of the fisheries of Crozet Islands. Fisheries Centre Research Reports, 23, 31.

[ece32677-bib-0041] Pruvost, P. , Martin, A. , Denys, G. , & Causse, R. (2011). Pecheker‐Simpa, a tool for fisheries management and ecosystem modelling. *Proceedings of the 1st international Science Symposium on the Kerguelen Plateau (Concarneau, 2010)*, The Kerguelen Plateau, Marine Ecosystem and Fisheries: 263–270 pp.

[ece32677-bib-0042] Ryan, P. G. , & Boix‐Hinzen, C. (1999). Consistent male‐biased seabird mortality in the Patagonian toothfish longline fishery. The Auk, 116(3), 851–854.

[ece32677-bib-0043] Shaffer, S. A. , Weimerskirch, H. , & Costa, D. P. (2001). Functional significance of sexual dimorphism in Wandering Albatrosses, *Diomedea exulans* . Functional Ecology, 15, 203–210. doi: 10.1046/j.1365‐2435.2001.00514.x

[ece32677-bib-0044] Skov, H. , & Durinck, J. (2001). Seabird attraction to fishing vessels is a local process. Marine Ecology Progress Series, 214, 289–298. doi: 10.3354/meps214289

[ece32677-bib-0045] Sugishita, J. , Torres, L. G. , & Seddon, P. J. (2015). A new approach to study of seabird‐fishery overlap: Connecting chick feeding with parental foraging and overlap with fishing vessels. Global Ecology and Conservation, 4, 632–644. doi: 10.1016/j.gecco.2015.11.001

[ece32677-bib-0046] Tew Kai, E. , Benhamou, S. , van der Lingen, C. D. , Coetzee, J. C. , Pichegru, L. , Ryan, P. G. , & Grémillet, D. (2013). Are Cape gannets dependent upon fishery waste? A multi‐scale analysis using seabird GPS‐tracking, hydro‐acoustic surveys of pelagic fish and vessel monitoring systems. Journal of Applied Ecology, 50, 659–670. doi: 10.1111/1365‐2664.12086

[ece32677-bib-0047] Tickell, W. L. N. (2000). Albatrosses. Mountfield, Sussex, UK: Pica Press.

[ece32677-bib-0048] Tixier, P. , Gasco, N. , Duhamel, G. , Viviant, M. , Authier, M. , & Guinet, C. (2010). Interactions of Patagonian toothfish fisheries with killer and sperm whales in the Crozet islands Exclusive Economic Zone: An assessment of depredation levels and insights on possible mitigation strategies. CCAMLR Science, 17, 179–195.

[ece32677-bib-0049] Torres, L. G. , Sagar, P. M. , Thompson, D. R. , & Phillips, R. A. (2013a). Scaling down the analysis of seabird‐fishery interactions. Marine Ecology Progress Series, 473, 275–289. doi: 10.3354/meps10071

[ece32677-bib-0050] Torres, L. G. , Sagar, P. M. , Thompson, D. R. , & Phillips, R. A. (2013b). Scale‐dependence of seabird‐fishery data analysis and management: Reply to Croxall et al. (2013). Marine Ecology Progress Series, 493, 301–304. doi: 10.3354/meps10600

[ece32677-bib-0051] Tuck, G. N. , Thomson, R. B. , Barbraud, C. , Delord, K. , Louzao, M. , Herrera, M. , & Weimerskirch, H. (2015). An integrated assessment model of seabird population dynamics: Can individual heterogeneity in susceptibility to fishing explain abundance trends in Crozet wandering albatross? Journal of Applied Ecology, 52, 950–959.

[ece32677-bib-0052] Votier, S. C. , Bearhop, S. , Witt, M. J. , Inger, R. , Thompson, D. , & Newton, J. (2010). Individual responses of seabirds to commercial fisheries revealed using GPS tracking, stable isotopes and vessel monitoring systems. Journal of Applied Ecology, 47, 487–497. doi: 10.1111/j.1365‐2664.2010.01790.x

[ece32677-bib-0053] Votier, S. C. , Furness, R. W. , Bearhop, S. , Crane, J. E. , Caldow, R. W. G. , Catry, P. , … Thompson, D. R. (2004). Changes in fisheries discard rates and seabird communities. Nature, 427, 727–730. doi: 10.1038/nature02315 14973483 10.1038/nature02315

[ece32677-bib-0054] Weimerskirch, H. (1995). Regulation of foraging trips and incubation routine in male and female wandering albatrosses. Oecologia, 102, 37–43. 28306805 10.1007/BF00333308

[ece32677-bib-0055] Weimerskirch, H. , Brothers, N. , & Jouventin, P. (1997). Population dynamics of wandering albatross *Diomedea exulans* and Amsterdam albatross *D. amsterdamensis* in the Indian Ocean and their relationships with long‐line fisheries: Conservation implications. Biological Conservation, 79, 257–270.

[ece32677-bib-0056] Weimerskirch, H. , Capdeville, D. , & Duhamel, G. (2000). Factors affecting the number and mortality of seabirds attending trawlers and long‐liners in the Kerguelen area. Polar Biology, 23, 236–249.

[ece32677-bib-0057] Weimerskirch, H. , Cherel, Y. , Delord, K. , Jaeger, A. , Patrick, S. C. , & Riotte‐Lambert, L. (2014). Lifetime foraging patterns of the wandering albatross: Life on the move!. Journal of Experimental Marine Biology and Ecology: “Charismatic marine mega‐fauna”, 450, 68–78. doi: 10.1016/j.jembe.2013.10.021

[ece32677-bib-0058] Weimerskirch, H. , Jouventin, P. , & Stahl, J.‐C. (1986). Comparative ecology of the six albatross species breeding on the Crozet Islands. Ibis, 128, 195–213. doi: 10.1111/j.1474‐919X.1986.tb02669.x

[ece32677-bib-0059] Weimerskirch, H. , Lallemand, J. , & Martin, J. (2005). Population sex ratio variation in a monogamous long‐lived bird, the wandering albatross. Journal of Animal Ecology, 74, 285–291. doi: 10.1111/j.1365‐2656.2005.00922.x

[ece32677-bib-0060] Weimerskirch, H. , Salamolard, M. , Sarrazin, F. , & Jouventin, P. (1993). Foraging strategy of wandering albatrosses through the breeding season: A study using satellite telemetry. The Auk, 110(2), 325–342.

[ece32677-bib-0061] Zuur, A. F. (2009). Mixed effects models and extensions in ecology with R, Statistics for biology and health. New York, NY: Springer [etc.].

