# Supplementary figures and images for: Behavioral responses to encounter of fishing boats in wandering albatrosses
Source: Ecol Evol. 2017 Apr 4;7(10):3335–47. doi: 10.1002/ece3.2677 (PMC5433987; doi:10.1002/ece3.2677)

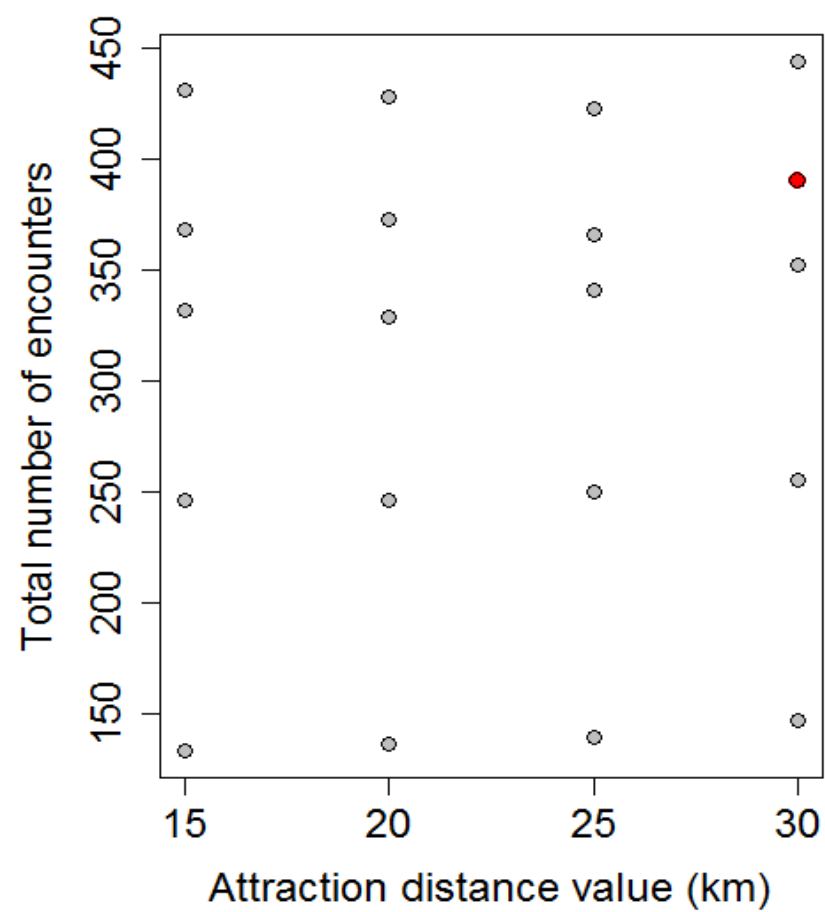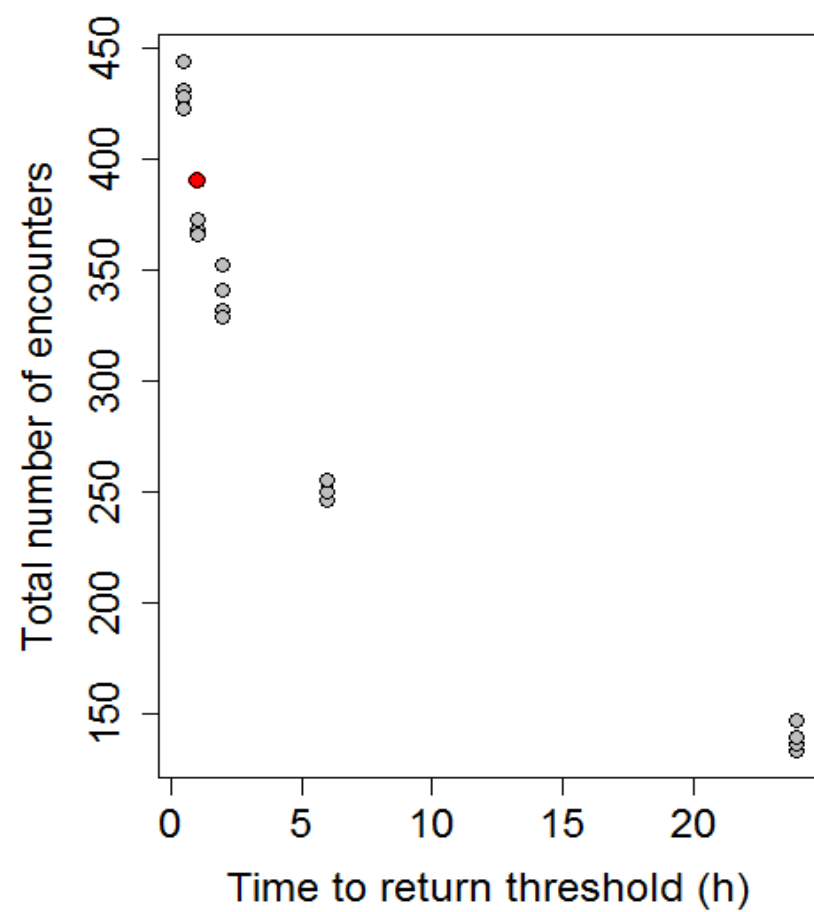

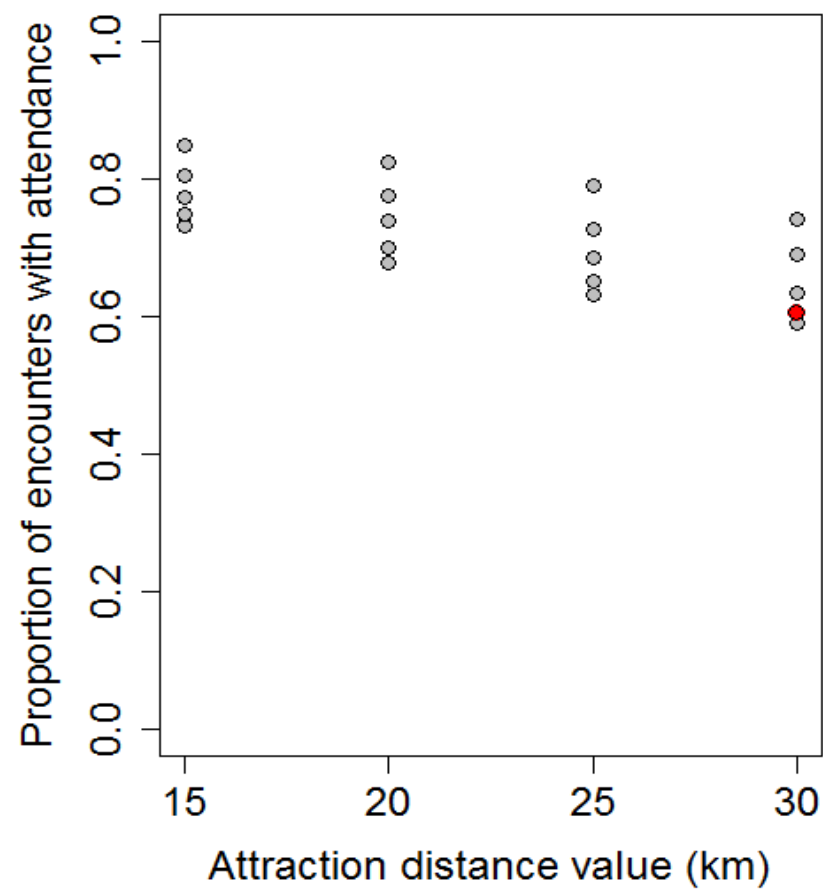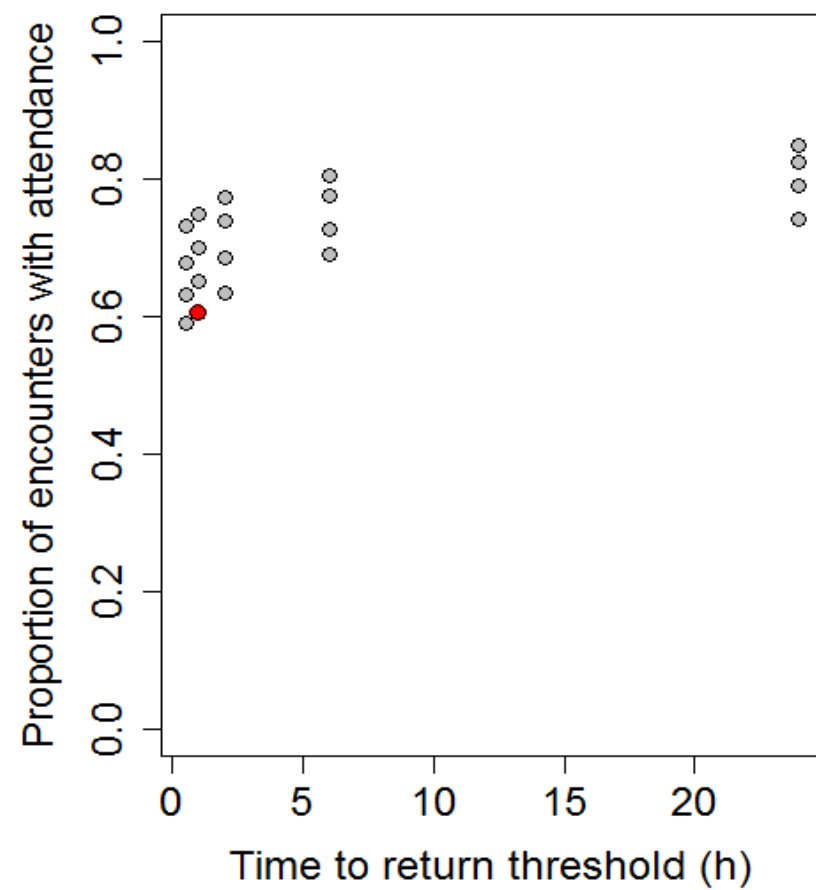

Supplement: Supplementary file 1 [file ECE3-7-3335-s001.pdf]
