# Supplementary material for: Behavioral responses to encounter of fishing boats in wandering albatrosses
Source: Ecol Evol. 2017 Apr 4;7(10):3335–47. doi: 10.1002/ece3.2677 (PMC5433987; doi:10.1002/ece3.2677)
Supplement: Supplementary file 2 [file ECE3-7-3335-s002.docx]

**Sensitivity Analysis**

**Methods**

We investigated the influence of the choice of parameter values for the attraction threshold and the time-to-return threshold on our results. In the main section, we set these to 30km and 1h, respectively. Here we re-run analyses for different combinations of values (15, 20, 25 and 30km; 30min, 1h, 2h, 6h, 24h). For each combination we report the number of encounters thus defined, and the proportion that was followed by attendance, as these are expected to largely vary with the encounter definition. Then we run GLMM for modelling the Encounter rate (model 1 in Table 2), the attraction probability (model 2) and the proportion of the whole encounter spent attending (model 4) the boat, with the same explanatory variables as the main section, to determine whether our conclusions on the relative effects of Sex, Age and Year are sensitive to the encounter definition. Models 3 and 5 are not expected to vary with this as we kept a constant attendance threshold of 3km. The average number of boats within 30km during encounter could not be accounted for in models with a time-to-return value of 24h, owing to a low variability of this variable.

**Results**

*Number of encounters and probability they contained attendance*

The total number of encounters appears to be mainly sensitive to the time-to-return value, suggestive of a come&go behaviour of wandering albatrosses. Our chosen value is in the upper range of value, reflecting on our choice to consider by default that if the bird leaves the direct attraction range, re-entering within this range should be considered a new encounter.


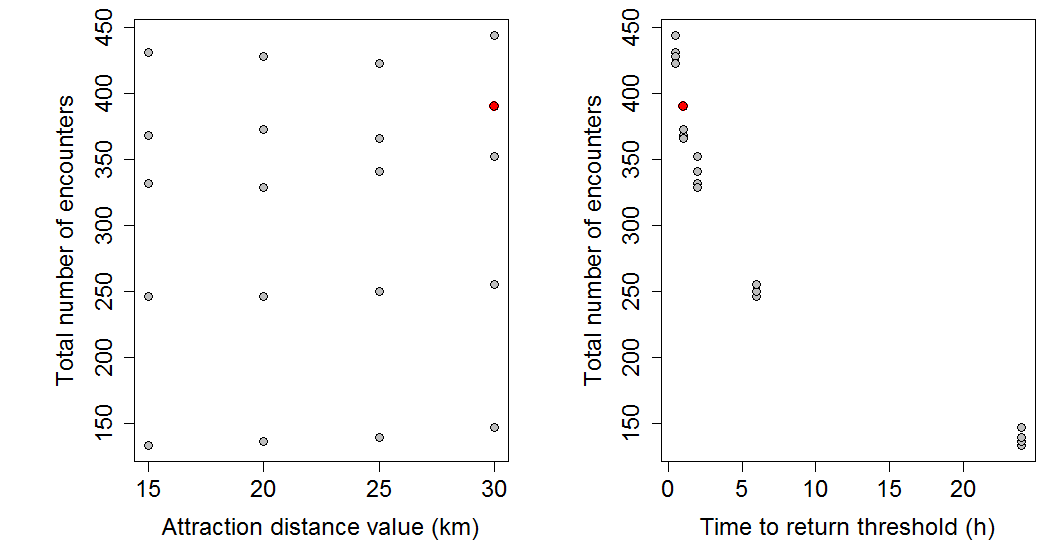


Figure 1: Sensitivity analyses on the number of encounters fitting the various definitions. The value reported in the main section is highlighted in red.

The proportion of encounters followed by attendance ranged from 59.0% to 85.0%. It increased with more restrictive definitions of encounters: in particular, birds were much more likely to be attracted once within closer and closer distances, consistent with results in (Collet et al., 2015). It is difficult to determine to what extent this is due to lower detection probability at larger distances, or simply reflect that shorter attraction threshold value introduce a bias by over-representing approaching birds. Modelling separately the detection probability and the attraction probability would require a completely different statistical modelling approach (i.e. probably Bayesian statistics) and the inclusion of additional explanatory variables to account for detection ability variation.

Time-to-return values weakly influenced this proportion, and only at low values.


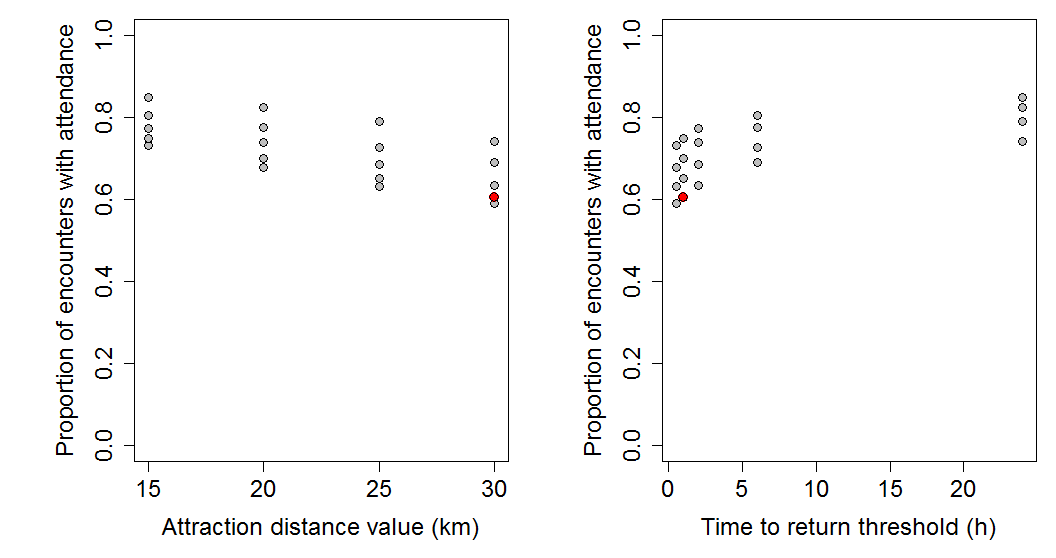


Figure 2: Sensitivity analyses on the attraction probability depending on the various encounter definitions. The value reported in the main section is highlighted in red.

*Sensitivity of age, sex and year effects*

Table 1: Proportion of models from different combinations of time-to-return and attraction threshold, in agreement with conclusions from main text (1h – 30km).

All cases of disagreement between models with different couples of parameter values were changes in statistical significance (i.e. variance of the effect value) rather than changes in the sign or strong quantitative changes in the effect mean value.

The encounter rate of boats was not influenced by the number of boats operating on the shelf for models 25km-24h and 30km-24h.

The average number of boats within 30km during the encounter had a significant positive effect on the probability to be attracted in 11 out of 16 models, a nearly significant effect in 3 models, but was not significant in 2 models. Significance of this variable decreased in models with short attraction threshold values (15km, sometimes 20km) and various time to return values (0.5, 1, 2 or 6h). This suggests that at these distances, birds are attracted anyway, no matter the number of boats (see also Figure 2).

The probability to be attracted was stronger when boats were operating, except for models 25km-24h and 30km-24h. In two models (20km-6h and 30km-24h), older males were significantly more attracted.

The 2013-year effect on the proportion of the encounter spent attending appeared sensitive to the definition of encounters: the 4 models with high attraction threshold (25, 30km) and large time-to-return values (24h, 6h), as well as models 30km-2h and 15km-0.5h did not retain significant year effects (although in three cases p<0.08).

The increased proportion of time spent attending boats during an encounter in females compared to males seemed robust, as this effect was significant or nearly significant (p-value when near significant 0.055, 0.061, 0.067, 0.075, 0.074) in all models. Moreover there was no clear pattern in parameter values when significance was not reached (20km-0.5h, 25km-1h, 25km-2h, 15km-6h, 20km-6h).

Finally, only half models supported the negative effect of the number of boats within 30km on the proportion of the encounter spent attending. These were models with high time-to-return values (8 models with values 2h or 6h), and models 25km-1h and 30km-1h.

**Conclusions:**

Our conclusions on the relative influence of age, sex and year appears very robust to the choice of attraction threshold and time to return values, even though these parameters can largely influence the number of encounters fitting the definition.

The attraction probability varied between 60% and 85% depending on the definitions used, with the upper values likely to be over-estimated by a priori discarding birds that ignored boats from distances >15km. Hence our conclusion that an important fraction of encounters were not followed by encounters seems robust too.

Finally, boat activity was found as an important predictor of the attraction probability and the proportion of time spent attending boats, for nearly all parameter combinations, confirming it as a central factor for the bird response to boats.
